# Relationships of Body Mass Index, Relative Fat Mass Index, and Waist Circumference with Serum Concentrations of Parameters of Chronic Inflammation

**DOI:** 10.3390/nu15122789

**Published:** 2023-06-18

**Authors:** Magdalena Sylwia Kamińska, Anna Lubkowska, Mariusz Panczyk, Ireneusz Walaszek, Szymon Grochans, Elżbieta Grochans, Anna Maria Cybulska

**Affiliations:** 1 Subdepartment of Long-Term Care and Palliative Medicine, Department of Social Medicine, Faculty of Health Sciences, Pomeranian Medical University in Szczecin, 48 Żołnierska St., 71-210 Szczecin, Poland; magdalena.kaminska@pum.edu.pl; 2 Department of Functional Diagnostics and Physical Medicine, Faculty of Health Sciences, Pomeranian Medical University in Szczecin, 54 Żołnierska St., 71-210 Szczecin, Poland; anna.lubkowska@pum.edu.pl; 3 Department of Education and Research in Health Sciences, Faculty of Health Sciences, Medical University of Warsaw, 14/16 Litewska St., 00-518 Warsaw, Poland; mariusz.panczyk@wum.edu.pl; 4 Department of Pediatric and Oncological Surgery, Urology and Hand Surgery, Pomeranian Medical University in Szczecin, 1 Unii Lubelskiej St., 72-252 Szczecin, Poland; irekwalaszek@gmail.com; 5 Department of Specialised Nursing, Faculty of Health Sciences, Pomeranian Medical University in Szczecin, 48 Żołnierska St., 71-210 Szczecin, Poland; szymon.grochans@pum.edu.pl; 6 Department of Nursing, Faculty of Health Sciences, Pomeranian Medical University in Szczecin, 48 Żołnierska St., 71-210 Szczecin, Poland; elzbieta.grochans@pum.edu.pl

**Keywords:** chronic inflammation, visceral adipose tissue, subcutaneous adipose tissue

## Abstract

(1) Background: Obesity in the perimenopausal period is associated with hormonal changes, lifestyle, and environment. In obesity, elevated levels of IL-6 and TNF-α and reduced levels of adiponectin are observed, and the associated chronic inflammation favors the development of cardiometabolic diseases. Therefore, the aim of our study was to assess the relationship between selected measures of obesity (BMI, WC, RFM, VAI, WHtR) and parameters of chronic inflammation (CRP, TNF-α, IL-6) in perimenopausal women. (2) Methods: The study involved 172 perimenopausal women. The methods used in this study were diagnostic surveys, anthropometric measurements, blood pressure measurements, and venous blood sampling. (3) Results: Preliminary multivariate linear regression analysis showed that CRP moderately positively correlated with IL-6 (β = 0.25; *p* = 0.001) and weakly negatively correlated with adiponectin (β = −0.23; *p* = 0.002). Similar associations were noted in preliminary multivariate linear regression analysis adjusted for age, menopausal status, and smoking status. Preliminary multivariate linear regression analysis also showed that BMI positively correlated with IL-6 (β = 0.16; *p* = 0.033). VAI weakly positively correlated with CRP (β = 0.25; *p* = 0.001) and negatively correlated with adiponectin (β = −0.43; *p* = 0.000). (4) BMI, WC, RFM, VAI, and WHtR are clearly related to selected parameters of chronic inflammation. Our study suggests that each of the anthropometric variables provides distinct information on metabolic processes associated with inflammatory parameters.

## 1. Introduction

Natural menopause, as defined by the World Health Organization (WHO), is the “permanent cessation of menstruation, resulting from the loss of ovarian follicular activity” [1]. Menopause is a critical stage in women’s aging and reproductive health. This is when significant changes in fat mass and fat distribution occur, along with dyslipidemia and neurodegeneration [2,3,4].

The Stages of Reproductive Aging Workshop (STRAW), held in 2001, proposed a nomenclature and consistent staging system for ovarian aging, including menstrual and qualitative hormonal criteria for each stage [5]. The STRAW staging system is widely regarded as a complex standard for characterizing reproductive aging during menopause. Accordingly, STRAW divided the adult female life into three periods: reproductive, the menopausal transition, and postmenopause. While premenopause is defined as the entire period of reproductive life before menopause, perimenopause is the time immediately before the last menstrual bleeding, during which menopausal (endocrine, biological, clinical) symptoms appear. Postmenopause is the time after the last menstruation (natural or surgical) [6]. Menopausal status is a key indicator of the endogenous hormonal environment in women. The menopausal transition is a time of many changes in health and quality of life. Menopause is a natural process. It affects every woman as she ages [7] and involves a variety of symptoms, including hot flashes, night sweats, palpitations, mood swings, insomnia, anxiety, depression, concentration problems, nervousness, headaches, states of tension, and tearfulness [8]. 

Cardiovascular disease (CVD) is a serious problem often faced by women entering menopause. The changing hormonal environment predisposes to cardiovascular disease due to the accumulation of risk factors, among them visceral obesity, dyslipidemia, impaired glucose homeostasis, hypertension, and non-alcoholic fatty liver disease [6,7].

Aging entails an increased risk of obesity [9] and non-communicable diseases (NCDs), including cardiovascular disease, type 2 diabetes, hypertension, and stroke [10]. A higher risk of non-communicable diseases is related to central obesity, increased waist circumference, and increased body mass index (BMI). During the perimenopausal period, women often suffer from undesired weight gain, which may be associated with the hormonal changes that then occur. Postmenopausal women may experience more severe changes in lean and fat body mass than men of a similar age [11].

A review of the literature indicates that visceral adipose tissue increases in middle age, unlike lean body mass, which appears to be reduced, possibly leading to sarcopenic obesity [12]. Additionally, in obesity, elevated levels of interleukin 6 (IL-6) and tumor necrosis factor alpha (TNF-α) [13] are observed. The related chronic inflammation promotes the development of cardiovascular disease, insulin resistance, and diabetes [14,15]. In obesity and diabetes, adiponectin (ADPN) levels are reduced, thus contributing to the deterioration of insulin resistance [16,17].

During the postmenopausal period, both obese and insulin-resistant women have reduced serum adiponectin levels [18], which may increase the incidence of metabolic syndrome (MetS), osteoporosis, and obesity [19]. The mechanism underlying elevated inflammatory markers in postmenopausal women is unclear, but it may be linked to the production of inflammatory markers by adipose tissue or the effect of inflammatory markers on insulin-stimulated glucose utilization. In addition, estradiol and progesterone regulate TNF-α, so the decline in hormone levels during menopause may affect the production of inflammatory markers [20]. It is worth noting that estradiol modulates cytokines, including IL-6, Interleukin 1 beta (IL-1β), and TNF-α [21]. According to some authors, postmenopausal status is more associated with increased levels of pro-inflammatory markers than premenopausal status [22,23], with IL-6 levels negatively correlating with estradiol levels during the menopausal transition [24]. Elevated levels of pro-inflammatory immune markers, such as IL-6 and C-reactive protein (CRP), affect cardiac health and metabolism [14,25], contributing to the risk of cardiovascular events in postmenopausal women [26,27]. 

It is also worth noting that menopause is associated with changes in the lipid profile. Postmenopausal women have higher levels of total cholesterol, triglycerides, and low-density lipoprotein cholesterol (LDL-C), as well as reduced levels of high-density lipoprotein cholesterol (HDL-C) [28]. Additionally, after menopause, the risk of MetS increases due to impaired glucose metabolism, weight gain, and central abdominal obesity [29,30]. Moreover, in menopausal women, waist to height ratio (WHtR) [31], waist to hip ratio (WHR) [32], and lipid accumulation product (LAP) [33] can predict cardiovascular disease and its risk factors [34].

## 2. Materials and Methods

A total of 172 perimenopausal women living in the West Pomeranian Voivodeship were invited to take part in the study.

The inclusion criteria for the study were: age 44–65, no history of menopausal hormone therapy (MHT), no previous psychiatric treatment, eating a normal diet based on Polish cuisine, no supplementation with micro- or macroelements. The exclusion criteria were: the use of MHT, psychiatric treatment, thyroid disease, cancer, refusal to participate in the study, and incorrectly completed documentation. 

The study was conducted in accordance with the Declaration of Helsinki after obtaining the approval of the Bioethics Committee of the Pomeranian Medical University in Szczecin (KB-0012/181/13). Recruitment was based on information posters in public places and advertisements in local newspapers. Each respondent gave informed written consent to participate in the study. 

This study is part of a larger project aimed at assessing the health status of perimenopausal women from the area of the West Pomeranian Voivodship. 

### 2.1. Research Project

The research was conducted in several stages. After obtaining written consent, the patients filled in a proprietary questionnaire concerning sociodemographic data (age, marital status, place of residence, education, employment status), stimulants (alcohol, caffeine, tobacco), and health (menstruation, inflammation, mental and cancer diseases, menopausal status). Menopausal status was classified as perimenopause (menopausal transition with irregular menstruation) and menopause (last menstruation at least 12 months prior to the study).

The next step was to take anthropometric measurements, measure blood pressure, and analyze fasting blood samples. 

### 2.2. Anthropometric Measurements

The next step was anthropometric measurements (waist and hip circumference, body weight, height).

- Waist and hip circumference were measured to the nearest 0.01 m using a flexible tape measure (SECA 711). Waist circumference (WC) was measured as the horizontal distance around the abdomen at the level of the navel. Hip circumference (HC) was measured as the horizontal distance across the two upper hip bones (ilia).
- Body weight and height were measured using a legalized medical scale with an integrated SECA 711 height meter in accordance with a standardized procedure with an accuracy of 0.1 kg and 0.1 cm, respectively. Participants stood with their backs straight, heels together, barefoot, in light clothing.

### 2.3. Indicators of Obesity 

In order to assess obesity in the women, the following were analyzed:

- body mass index (BMI) was calculated by the formula: BMI = body weight (kg)/height (m)^2^. BMI was divided into the following categories according to the Centers for Disease Control and Prevention (CDC): underweight (BMI < 18.5), normal weight (BMI = 18.5–24.9), overweight (BMI = 25.0–29.9) and obesity (BMI ≥ 30) [35];
- waist circumference (WC): abdominal (central) obesity was diagnosed when WC ≥ 80 cm (for women in Europe) [36];
- relative fat mass (RFM) index was calculated by the formula: RFM = 76 − (20 × (height (m)/waist circumference (m))). The result of the RFM equation is written as a percentage—it reflects the approximate fat content in the human body. For an obese person, the percentage of body fat (PBF) is ≥ 32% for women [37];
- visceral adipose index (VAI) was calculated by the formula: VAI = [WC/39.68 + (1.88 × BMI)] × (TG/1.03) × (1.31/HDL). This index helps to assess visceral adipose dysfunction and cardiometabolic risk [37].
- waist to height ratio (WHtR) was calculated according to the formula: WHtR = (WC) (cm)/height (cm).

### 2.4. Blood Pressure Measurement

The Korotkoff sound technique was used to measure blood pressure. Measurements were taken in accordance with the recommendations of the American Heart Association [38] and the Polish Cardiac Society [39]. The examination was performed on the right upper limb artery in a sitting position. The correct position of the body, a period of rest, an appropriate-size cuff, and the minimization of external factors affecting blood pressure (smoking and ingestion of caffeine-containing products) were ensured prior to blood pressure measurement [40].

### 2.5. Blood Collection

Blood was drawn fasting between 7:00 and 9:30 a.m. after a 10 min rest, in a sitting position, using the Vacutainer system by qualified nurses. Blood sampling was carried out in accordance with the rules and procedures for collecting, storing, and transporting biological material. Biochemical analysis was carried out in a certified laboratory of the Pomeranian Medical University in Szczecin—standard commercial methods were used. 

Biochemical parameters—insulin, glucose, glycated hemoglobin (HbA1c), total cholesterol (TC), HDL, LDL, triglycerides (TG), and CRP—were measured. 

Serum levels of IL-6, TNF-α, and adiponectin were determined by an enzyme-linked immunosorbent assay (Immundiagnostik, Bensheim, Germany), while hs-CRP by immunonephelometry (Behring Nephelometer II, Dade Behring, Marburg, Germany) [41].

### 2.6. Determination of IL-6 Level

To determine selected serum inflammatory markers, we collected blood into serum separation tubes. Serum IL-6 level was measured by the enzyme-linked immunosorbent assay (ELISA) (DRG, Germany). The sensitivity of the IL-6 assay was 2 pg/mL, the intra- and interassay CV values were 4.2% and 4.4%, respectively [41].

### 2.7. Classification of Respondents

The study recruited 200 perimenopausal women aged 45 to 65 representing the general population of the West Pomeranian Voivodeship in northwestern Poland. Ultimately, 172 respondents who met all inclusion criteria were included in the study (completion rate: 86%). 

The size of the study sample was determined based on statistical data on the size of the population of women aged 45–64 in the West Pomeranian Voivodeship in 2020 [42]. The confidence level was set at 95%, the maximum error at 7%, and the estimated fraction size at 0.5. 

The women were divided into groups according to the following variables:

(1) Menopausal status [6]:
  - perimenopausal women—immediately before menopause, with endocrinological, biological, and clinical symptoms of approaching menopause;
  - postmenopausal women—the last menstruation at least 12 months before the study.
(2) Metabolic Syndrome (MetS): based on the latest criteria proposed by the International Diabetes Federation (IDF) and the modified National Cholesterol Education Program-Adult Treatment Panel III (NCEP-ATP III) [43]; MetS is diagnosed in women if three out of the following five risk factors are present:
  - WC ≥ 80 cm or treatment for this lipid abnormality;
  - TG > 150 mg/dL (1.7 mmol/L) or treatment for this lipid abnormality;
  - HDL < 50 mg/dL (1.3 mmol/L) or treatment for this lipid abnormality;
  - elevated blood pressure (BP): systolic blood pressure (SBP) ≥ 130 or diastolic blood pressure (DBP) ≥ 85 mmHg or treatment for hypertension;
  - fasting plasma glucose (FPG) ≥ 100 mg/dL (5.6 mmol/L) or a diagnosis of type 2 diabetes. If FPG > 100 mg/dL (5.6 mmol/L), it is strongly recommended to perform the oral glucose tolerance test (OGTT).

The women who did not meet the above criteria for MetS diagnosis but had at least two components of MetS were defined as having pre-metabolic syndrome (pre-MetS) [44].

(3) Obesity [35,36]:
  - abdominal obesity diagnosed in women if WC ≥ 80 cm;
  - general obesity diagnosed in women if BMI ≥ 30 kg/m^2^.

### 2.8. Statistical Analysis 

The data on CRP, TNF-α, IL-6, and adiponectin were not distributed normally and were therefore log transformed for further analyses. We calculated Pearson’s correlations between measures of obesity and between selected parameters of chronic systemic inflammation. Additionally, we calculated partial correlation coefficients between inflammatory parameters adjusted for age, current smoking status, and menopausal status. 

In the first model, all parameters of chronic inflammation were mutually adjusted. Multivariate linear regression analysis was performed to assess the relationships of BMI, WC, RFM, VAI, WHtR to CRP, TNF-α, IL-6, and adiponectin, adjusted for obesity (BMI < 30.0 kg/m^2^, and BMI ≥ 30.0 kg/m^2^), smoking status (currently smoking, non-smoking), and MetS (pre-MetS, Mets, no-MetS).

When reporting the results from the linear regression models, β-coefficients were considered weak (β ≤ 0.3), moderate (β > 0.3 to ≤0.6), or strong (β > 0.6). 

All statistical calculations were performed using Statistica v. 13.3 (TIBCO Software Inc, Palo Alto, CA, United States). For all analyses, a two-tailed *p* < 0.05 was considered statistically significant.

## 3. Results

### 3.1. Subject Characteristics

Characteristics of the study sample are shown in Table 1. The study involved 172 women, whose mean age was 54.49 years (SD = 4.99). The vast majority of the respondents were postmenopausal women (74.42%). 

Statistically significant differences between the groups defined by menopausal status were noted for age (*p* < 0.001) and the levels of LDL (*p* = 0.043) and IL-6 (*p* = 0.048). Postmenopausal women had significantly lower LDL levels and higher IL-6 levels than their premenopausal counterparts (Table 1). For the rest of variables, there were no statistically significant differences between the groups. The general characteristics of the population studied is included in Table S1.

### 3.2. Interrelationships between Selected Parameters of Chronic Systemic Inflammation

We analyzed the interrelationships between selected parameters of chronic systemic inflammation. Preliminary multivariate linear regression analysis showed that CRP moderately positively correlated with IL-6 (β = 0.25; *p* = 0.001) and weakly negatively correlated with adiponectin (β = −0.23; *p* = 0.002). Similar associations were noted in preliminary multivariate linear regression analysis adjusted for age, menopausal status, and smoking status. No correlations were found between TNF-α and CRP or between IL-6 and adiponectin (Table 2).

### 3.3. Relationships of BMI, WC, RFM, VAI, and WHtR with Selected Parameters of Chronic Systemic Inflammation

We analyzed the relationships between anthropometric parameters (BMI, WC, RFM, VAI, WHtR) and selected parameters of chronic systemic inflammation.

Preliminary multivariate linear regression analysis showed that BMI positively correlated with IL-6 (β = 0.16; *p* = 0.033). VAI weakly positively correlated with CRP (β = 0.25; *p* = 0.001) and negatively correlated with adiponectin (β = −0.43; *p* = 0.000). 

Multivariate linear regression analysis adjusted for age, menopausal status, and smoking status showed that VAI weakly positively correlated with CRP (β = 0.25; *p* = 0.001) and negatively correlated with adiponectin (β = −0.43; *p* < 0.001). No other statistically significant relationships between the anthropometric and inflammatory parameters were observed (Table 3).

### 3.4. Relationships between Anthropometric Parameters (BMI, WC, RFM, VAI, WHtR) and Selected Parameters of Chronic Systemic Inflammation in the Subgroups Defined by BMI, Smoking Status, and MetS

We analyzed the relationships between selected anthropometric parameters (BMI, WC, RFM, VAI, WHtR) and the parameters of chronic systemic inflammation in the subgroups defined by BMI, smoking status, and MetS.

Next, we conducted analysis stratified by obesity (BMI ≥ 30.0 kg/m^2^) adjusted for age and menopausal status (Table 4). In subjects with BMI < 30.0 kg/m^2^, VAI positively correlated with CRP (β = 0.19; *p* = 0.035) and moderately negatively correlated with adiponectin (β = −0.42; *p* < 0.001). In subjects with BMI ≥ 30.0 kg/m^2^, VAI negatively correlated with adiponectin (β = −0.29; *p* = 0.033). There were no statistically significant correlations of BMI, WC, RFM, and WHtR with other inflammatory parameters (Table 4). 

In the group of current non-smokers, BMI positively correlated with IL-6 (β = 0.18; *p* = 0.048), RFM negatively correlated with TNF-α (β = −0.21; *p* = 0.021), and VAI moderately negatively correlated with adiponectin (β = −0.37; *p* < 0.001). By comparison, in current smokers, WC, RFM, and WHtR showed a moderate positive correlation with TNF-α (β = 0.39; *p* = 0.008; β = 0.31; *p* = 0.030; β = 0.32; *p* = 0.034, respectively). In addition, there was a moderate positive correlation between VAI and IL-6 (β = 0.34; *p* = 0.012), and a moderate negative correlation between VAI and adiponectin (β = −0.47; *p* < 0.001) (Table 4). 

In the groups defined by MetS, a moderate negative correlation was observed between VAI and adiponectin (β = −0.47; *p* = 0.001). No statistically significant correlations of BMI, WC, RFM, and WHtR with other inflammatory parameters were found in the no-MetS, MetS, and pre-MetS groups (Table 4).

### 3.5. Relationships between Plasma Adiponectin Levels and Cardiovascular Risk Factors

We analyzed the relationships between inflammatory parameters and cardiovascular risk factors. 

In the group of premenopausal women, we observed statistically significant positive correlations between CRP and FSG (r = 0.38; *p* = 0.011) and between insulin (r = 0.57; *p* < 0.001) and HOMA-IR (r = 0.52; *p* < 0.001). Furthermore, adiponectin statistically significantly negatively correlated with FSG (r = −0.47; *p* = 0.001) and insulin (r = −0.46; *p* = 0.002), and HOMA-IR (r = −0.56; *p* < 0.001) and statistically significantly positively correlated with HDL (r = 0.53; *p* < 0.001). There were no statistically significant correlations between the rest of inflammatory parameters and selected cardiovascular risk factors (Table 5). 

In the group of postmenopausal women, CRP statistically significantly positively correlated with SBP (r = 0.22, *p* = 0.014), FSG (r = 0.42, *p* < 0.001), insulin (r = 0.33, *p* < 0.001), and HOMA-IR (r = 0.46, *p* < 0.001). TNF-α statistically significantly positively correlated with LDL (r = 0.20, *p* = 0.020), and IL-6 statistically significantly positively correlated with BMI (r = 0.20, *p* = 0.025), FSG (r = 0.18, *p* = 0.047), HOMA-IR (r = 0.20, *p* = 0.022), and CRP (r = 0.20, *p* = 0.025). Adiponectin statistically significantly negatively correlated with FSG (r = −0.28, *p* = 0.002), insulin (r = −0.45, *p* < 0.001), and HOMA-IR (r = −0.49, *p* < 0.001), and statistically significantly positively correlated with HDL (r = 0.53, *p* < 0.001). No statistically significant correlations were found between the other inflammatory parameters and selected cardiovascular risk characteristics (Table 5).

In the whole study sample, CRP statistically significantly positively correlated with SBP (r = 0.23, *p* = 0.003), FSG (r = 0.41, *p* < 0.001), insulin (r = 0.39, *p* < 0.001), and HOMA-IR (r = 0.48, *p* < 0.001), and statistically significantly negatively correlated with HDL (r = 0.39, *p* < 0.001). In addition, there was a statistically significant positive correlation between IL-6 and BMI (r = 0.16, *p* = 0.033), FSG (r = 0.20, *p* = 0.010), HOMA-IR (r = 0.20, *p* = 0.009), and CRP (r = 0.16, *p* = 0.039). Additionally, in this group adiponectin statistically significantly negatively correlated with FSG (r = −0.32, *p* < 0.001), insulin (r = −0.45, *p* < 0.001), HOMA-IR (r = −0.51, *p* < 0.001), and CRP (r = −0.15, *p* = 0.045), and statistically significantly positively correlated with HDL (r = 0.48, *p* < 0.001) (Table 5). 

## 4. Discussion

### 4.1. Interrelationships between Selected Parameters of Chronic Systemic Inflammation

In our study, the groups defined by menopausal status statistically significantly differed in terms of age, as well as LDL and IL-6 levels. Postmenopausal women had substantially lower LDL levels and higher IL-6 levels than premenopausal women. For the other variables, there were no statistically significant differences between the groups. Furthermore, CRP moderately positively correlated with IL-6 and weakly negatively correlated with adiponectin. Similar correlations were noticed in preliminary multivariate linear regression analysis adjusted for age, menopausal status, and smoking status. No relationships were found between TNF-α and CRP or between IL-6 and adiponectin. 

Although many studies show no association between menopausal status and CRP levels [20,45,46,47], Metcalf et al. [48] have provided evidence that menopausal status affects several markers of inflammation. Since, in their research, IL-6 levels were significantly higher during the late transition compared to the premenopausal period, these authors have concluded that the changes intrinsically linked to the menopausal transition (e.g., hormonal changes) may affect inflammatory markers. Similar results were obtained by Cioffi et al. [23], who noted that serum IL-6 levels were higher in postmenopausal women. Taleb-Belkadi et al. [22], on the other hand, reported that TNF-α and IL-1α were higher in postmenopausal women, while CRP levels were elevated in both peri- and postmenopausal women. 

According to Sites et al. [49], TNF-α was higher in postmenopausal women compared to premenopausal women. IL-6 and CRP did not differ depending on menopausal status. In both premenopausal and postmenopausal women, CRP positively correlated with total body fat. CRP correlated with abdominal fat only in postmenopausal women and negatively correlated with insulin and glucose levels in both premenopausal and postmenopausal women. 

As stated by Razmjou et al. [47], TNF-α levels increase in the peri- and postmenopausal periods, while the levels of IL-6 and IL-1β do not change. In addition, Lee et al. [20] have confirmed a statistically significant increase in adiponectin levels in pre- and postmenopausal women. Moreover, an increase in visceral (or intra-abdominal) fat was negatively correlated with changes in adiponectin. This finding suggests that an increase in visceral obesity during menopause has an adverse effect on the levels of adiponectin and inflammatory markers. Yasui et al. [24] noted that the concentration of IL-6 was weakly positively correlated with age. Postmenopausal women who were five years post menopause showed a significant increase in interleukin-2 (IL-2). Serum estradiol levels significantly negatively correlated with IL-6 and weakly negatively correlated with IL-2, interleukin-8 (IL-8), and granulocyte-macrophage colony-stimulating factor (GM-CSF). Vural et al. [50], on the other hand, reported that postmenopausal women, compared to premenopausal women, had elevated levels of TNF-α, IL-beta, and interleukin-10 (IL-10).

### 4.2. Relationships of BMI, WC, RFM, VAI, and WHtR with Selected Parameters of Chronic Systemic Inflammation 

Preliminary multivariate linear regression analysis in our study showed that BMI was positively correlated with IL-6. Moreover, VAI was observed to be weakly positively correlated with CRP and negatively correlated with adiponectin. In addition, multivariate linear regression analysis adjusted for age, menopausal status, and smoking status showed that VAI was weakly positively correlated with CRP and negatively correlated with adiponectin. No other statistically significant relationships were observed between the anthropometric (BMI, WC, RFM, VAI, and WHtR) and inflammatory parameters.

The linking mechanism and the complications between obesity and inflammation are well-documented in the literature, which indicates that the excess of macronutrients in the adipose tissues stimulates them to release inflammatory mediators such as TNF-α and IL-6 and reduces the production of adiponectin, predisposing to a pro-inflammatory state and oxidative stress. The increased level of IL-6 stimulates the liver to synthesize and secrete CRP. Inflammation is a risk factor for developed cardiovascular diseases including metabolic syndrome, insulin resistance, and diabetes mellitus [51,52].

Additionally, a review of the literature indicates that the correlations of VAT, BMI, and WC with hs-CRP are definitely stronger in women than in men [53], which is mainly due to increased estrogen production in adipose tissue. 

In a study by Park et al. [54], overall serum concentrations of CRP, TNF-α, and IL-6 were significantly correlated with body weight, BMI, WC, hip circumference, and WHR. In obese subjects, CRP and IL-6 significantly correlated with BMI, WC, and VAT. Multivariate regression analysis revealed that CRP was significantly related to BMI, while IL-6 was significantly associated with visceral adiposity in obese subjects. 

Additionally, Piché et al. [55] informed that hs-CRP and IL-6 were significantly associated with anthropometric and metabolic variables, including VAT and SAT, SBP, DBP, TG, HDL, and insulin sensitivity. Women with higher hs-CRP levels had worse metabolic risk profiles, including abdominal obesity, higher TG and lower HDL levels, and lower insulin sensitivity compared to women with lower hs-CRP levels.

In a study by Panagiotakos et al. [53], all inflammatory markers (CRP, TNF-α, serum amyloid A (SAA), white blood cells and IL-6 were related to BMI, WC, and WHR in both sexes. Moreover, models that included WC or hip circumference as an independent variable had higher explanatory power than models with BMI, especially in women, even after adjusting for age and various other potential confounders. In the case study of Thorand et al. [56], it was observed that fat mass, BMI, WC, and WHR were strongly correlated with markers of systemic inflammation (CRP, SAA, fibrinogen (FIB), IL-6) in both women and men.

Judkin et al. [57] reported that the levels of CRP, IL-6, and TNF-α were statistically significantly related to measures of obesity (BMI, WHR). In addition, CRP levels were significantly associated with IL-6 and TNF-α levels, as well as HOMA-IR, SBP, HDL, and TG. These data suggest that adipose tissue is an important determinant of low levels of chronic inflammation, as reflected in IL-6, TNF-α, and CRP levels. 

Schlecht et al. [58] informed that VAT, SAT, BMI, and WC were significantly associated with hs-CRP. Additionally, BMI was negatively correlated with adiponectin. Similar results were obtained among healthy adults, where VAT, SAT, BMI, and WC positively correlated with CRP [59,60,61]. While some studies have shown a comparable association of VAT and SAT with CRP [62], in others the relationship between CRP and VAT [55,60] or SAT was stronger [63].

In the analysis by Schlecht et al. [57], all anthropometric variables showed stronger correlations with hs-CRP in current smokers than in non-smokers. Shielts et al. [64] confirm that cigarette smoking is associated with increased CRP levels. There are few studies concerning the relationship between obesity and CRP, taking into account smoking status [59]. A study by Forouhi et al. [59] showed that obesity measures and CRP levels were significantly correlated in both South Asian and European residents. In addition, strong correlations were noted between CRP and central obesity measures (WC and visceral fat area) in South Asians. In Europeans, BMI and PBF were significantly associated with CRP. In regression analysis adjusted for age, sex, and smoking, CRP levels were significantly correlated with fasting insulin and lipid levels in both ethnic groups. This means that obesity (especially VAT) is a key promoter of low-grade chronic inflammation. 

Marques-Vidal et al. [65] found no differences in IL-1β levels between healthy respondents and participants with elevated obesity markers. In addition, multivariate regression analysis revealed a negative correlation between PBF and IL-1β. Participants with high PBF or abdominal obesity had higher levels of IL-6, but no independent association was found between IL-6 levels and obesity markers. Respondents with abdominal obesity had higher levels of TNF-α, and positive correlations were found between TNF-α and BMI in women. Obese participants had higher levels of hs-CRP, and these differences persisted after multivariate adjustment. Similarly, positive correlations were found between hs-CRP levels and all obesity markers analyzed in the study.

## 5. Conclusions

BMI, WC, RFM, VAI, and WHtR are clearly related to selected parameters of chronic inflammation. Our study suggests that each of the anthropometric variables provides distinct information on metabolic processes associated with inflammatory parameters.

While BMI was an indicator of elevated IL-6 levels, VAI was a significant indicator of decreased levels of adiponectin. Subgroup analyses revealed that BMI, MetS, and smoking were factors modifying the relationships between obesity measures and inflammatory parameters. 

## 6. Limitation and Strength 

The strength of our study is expressed when examining the relationship between selected measures of obesity (BMI, WC, RFM, VAI, WHtR) and parameters of chronic inflammation (CRP, TNF-α, IL-6) in perimenopausal healthy women. Another strength of this study was examining the interrelationships between selected parameters of chronic systemic inflammation, the relationships between anthropometric parameters (BMI, WC, RFM, VAI, WHtR) and selected parameters of chronic systemic inflammation, as well as the relationships between selected anthropometric parameters (BMI, WC, RFM, VAI, WHtR) and the parameters of chronic systemic inflammation in the subgroups defined by BMI, smoking status, and MetS, and the relationships between plasma adiponectin levels and cardiovascular risk factors in perimenopausal healthy women. As far as we know, this is the first study that has assessed the above.

The limitation of our study is the small sample size, which may result in insufficient statistical power to detect specific relationships. Another limitation was the nature of our study design, which did not allow the assessment of cause-effect relationships between selected measures of obesity (BMI, WC, RFM, VAI, WHtR) and parameters of chronic inflammation (CRP, TNF-α, IL-6) in perimenopausal healthy women. Moreover, our analyzes may not reflect the actual long-term mean serum concentrations of chronic inflammation parameters in perimenopausal healthy women as they were based on a single laboratory measurement.

## Figures and Tables

**Table 1:** Characteristics of the participants.

Variables	Premenopausal (*n* = 44)		Postmenopausal (*n* = 128)		All (*n* = 172)		t_df_ = 233	*p*-Value *
	M	SD	M	SD	M	SD		
Age (years)	48.70	2.85	56.48	3.90	54.49	4.99	−12.152	<0.001
Menopausal age (years)	-	-	48.54	4.46	48.54	4.46	-	-
Time since menopause (years)	-	-	7.94	5.09	7.94	5.09	-	-
Body mass (kg)	75.86	19.08	75.05	13.52	75.26	15.14	0.301	0.7639
BMI (kg/m^2^)	28.44	6.72	28.66	5.38	28.60	5.73	−0.217	0.829
WC (cm)	88.43	12.41	90.20	13.11	89.75	12.92	−0.785	0.433
WHtR	0.54	0.08	0.56	0.09	0.55	0.08	−1.085	0.279
RFM	38.41	5.13	39.33	5.59	39.09	5.48	−0.961	0.338
VAI	1.60	1.89	1.56	1.08	1.57	1.33	0.196	0.845
HbA1c (%)	5.56	0.99	5.64	1.02	5.62	1.01	−0.467	0.641
FBG (mg/dL)	92.54	31.63	95.17	38.37	94.46	36.78	−0.407	0.685
Insulin (µIU/L)	9.56	6.26	10.04	5.99	9.91	6.05	−0.454	0.650
SBP (mmHg)	122.32	16.86	124.31	19.12	123.80	18.54	−0.614	0.540
Cortisol (µg/dL)	16.48	7.41	14.85	6.12	15.27	6.49	1.442	0.151
DBP (mmHg)	78.52	10.63	77.15	10.69	77.50	10.66	0.737	0.462
Total cholesterol (mg/dL)	215.90	32.05	204.36	38.23	207.31	37.01	1.797	0.074
LDL (mg/dL)	69.13	18.40	62.91	17.09	64.50	17.59	2.042	0.043
HDL (mg/dL)	123.87	35.03	120.48	33.01	121.35	33.46	0.578	0.564
Triglycerides (mg/dL)	108.68	81.65	105.61	50.41	106.40	59.71	0.294	0.769
Adiponectin (ng/mL)	11,328.03	5557.73	10,866.76	6476.75	10,984.76	6242.00	0.422	0.674
CRP (mg/dL)	3.74	10.04	3.24	7.49	3.37	8.19	0.346	0.730
TNF-α (pg/mL)	4.22	5.29	5.37	8.18	5.08	7.56	−0.871	0.385
IL-6 (pg/mL)	16.25	21.71	64.63	160.43	52.26	140.30	−1.990	0.048
HOMA-IR	2.45	2.78	2.48	2.10	2.47	2.28	−0.054	0.957

BMI—body mass index, WC—waist circumference, WHtR—waist-to-height ratio, RFM—relative fat mass, VAI—visceral adiposity index, HbA1c—glycated hemoglobin, FBG—fasting blood glucose, SBP—systolic blood pressure, DBP—diastolic blood pressure, LDL—low density lipoprotein, HDL—high-density lipoprotein, CRP—C-reactive protein, TNF-α—tumor necrosis factor alpha, IL-6—Interleukin-6, HOMA-IR—homeostasis model assessment of insulin resistance, *—Student’s *t*-test, M—mean, SD—standard deviation, *N*—whole cohort size, *n*—number, *p*—significance level.

**Table 2:** Interrelationships between selected parameters of chronic systemic inflammation.

Independent Variable	Log CRP		Log TNF-α		Log IL-6	
	β	*p*	β	*p*	β	*p*
Log CRP *	-	-	0.09	0.237	0.25	0.001
Log CRP **	-	-	0.11	0.154	0.24	0.002
Log TNF-α *	0.09	0.237	-	-	0.11	0.143
Log TNF-α **	0.11	0.154	-	-	0.11	0.136
Log IL-6 *	0.25	0.001	0.11	0.143	-	-
Log IL-6 **	0.23	0.002	0.12	0.136	-	-
Log adiponectin *	−0.23	0.002	−0.01	0.901	−0.03	0.708
Log adiponectin **	−0.23	0.003	−0.01	0.879	−0.02	0.776

β—standardized regression coefficient, * unadjusted regression model, ** regression model adjusted for age, menopausal status, and smoking, CRP—C-reactive protein, TNF-α—tumor necrosis factor alpha, IL-6—Interleukin-6.

**Table 3:** Relationships of BMI, WC, RFM, VAI, and WHtR with selected parameters of chronic systemic inflammation.

Independent Variable	BMI		WC		RFM		VAI		WHtR	
	β	*p*	β	*p*	β	*p*	β	*p*	β	*p*
Log CRP *	0.06	0.442	0.02	0.798	0.05	0.533	0.25	0.001	0.04	0.642
Log CRP **	0.04	0.648	0.00	0.976	0.03	0.691	0.25	0.001	0.02	0.807
Log TNF-α *	−0.05	0.534	0.01	0.923	−0.09	0.263	0.06	0.413	−0.04	0.635
Log TNF-α **	−0.04	0.565	0.01	0.914	−0.08	0.282	0.07	0.365	−0.03	0.661
Log IL-6 *	0.16	0.033	0.07	0.349	0.10	0.180	0.07	0.379	0.10	0.176
Log IL-6 **	0.15	0.058	0.05	0.483	0.09	0.260	0.06	0.417	0.09	0.257
Log adiponectin *	0.05	0.502	0.01	0.933	−0.03	0.701	−0.43	0.000	−0.01	0.938
Log adiponectin **	0.06	0.453	0.01	0.873	−0.02	0.761	−0.43	<0.001	0.00	0.995

β—standardized regression coefficient, * unadjusted regression model, ** regression model adjusted for age, menopausal status, and smoking, BMI—body mass index, WC—waist circumference, RFM—relative fat mass, VAI—visceral adiposity index, WHtR— waist-to-height ratio, CRP—C-reactive protein, TNF-α—tumor necrosis factor alpha, IL-6—Interleukin-6.

**Table 4:** Relationships of BMI, WC, RFM, VAI, and WHtR with selected parameters of chronic systemic inflammation in the subgroups defined by BMI, smoking status, and MetS.

Independent Variable	BMI < 30.0 kg/m^2^ (*n* = 116)										BMI ≥ 30.0 kg/m^2^ (*n* = 56)									
	BMI		WC		RFM		VAI		WHtR		BMI		WC		RFM		VAI		WHtR	
	β	*p*	β	*p*	β	*p*	β	*p*	β	*p*	β	*p*	β	*p*	β	*p*	β	*p*	β	*p*
Log CRP	0.00	0.979	−0.04	0.730	−0.02	0.835	0.19	0.035	−0.03	0.0793	−0.00	0.987	0.00	0.980	0.06	0.707	0.12	0.398	0.03	0.868
Log TNF-α	−0.08	0.423	−0.02	0.840	−0.09	0.334	0.06	0.450	−0.05	0.603	−0.04	0.803	0.03	0.821	−0.12	0.431	0.01	0.967	−0.05	0.741
Log IL-6	0.19	0.052	0.08	0.436	0.12	0.227	0.02	0.822	0.12	0.215	0.05	0.734	0.00	0.992	0.02	0.901	0.06	0.653	0.01	0.932
Log adiponectin	−0.02	0.865	−0.06	0.551	−0.09	0.377	−0.42	<0.001	−0.06	0.571	0.12	0.421	0.13	0.396	0.10	0.490	−0.29	0.033	0.09	0.550
	**Current non−smoking (*n* = 132)**										**Current smoking (*n* = 40)**									
Log CRP	0.04	0.686	−0.01	0.938	0.01	0.932	0.14	0.122	−0.00	0.959	−0.00	0.986	0.09	0.539	0.15	0.297	0.17	0.176	0.13	0.412
Log TNF-α	−0.11	0.202	−0.13	0.148	−0.21	0.021	0.10	0.254	−0.16	0.071	0.09	0.618	0.39	0.008	0.31	0.030	−0.10	0.395	0.32	0.034
Log IL-6	0.18	0.048	0.05	0.583	0.11	0.235	−0.05	0.587	0.11	0.247	0.03	0.863	0.27	0.076	0.27	0.079	0.34	0.012	0.25	0.123
Log adiponectin	0.07	0.466	−0.03	0.771	−0.08	0.368	−0.37	<0.001	−0.06	0.497	0.05	0.808	0.14	0.344	0.20	0.170	−0.47	0.001	0.20	0.196
	**No−MetS (*n* = 80)**										**Pre−MetS/MetS (*n* = 92)**									
Log CRP	−0.04	0.712	−0.08	0.516	−0.07	0.588	0.07	0.569	−0.05	0.702	0.07	0.533	0.03	0.801	0.03	0.781	0.05	0.649	0.02	0.865
Log TNF-α	−0.12	0.310	0.03	0.823	−0.11	0.381	0.14	0.253	−0.05	0.677	−0.02	0.877	−0.03	0.782	−0.10	0.377	0.07	0.443	−0.05	0.664
Log IL-6	0.10	0.424	0.11	0.387	0.12	0.355	0.10	0.417	0.12	0.346	0.20	0.067	−0.01	0.945	0.04	0.712	−0.05	0.615	0.04	0.713
Log adiponectin	0.10	0.380	0.02	0.886	−0.03	0.816	−0.19	0.088	−0.04	0.718	0.07	0.519	0.05	0.655	0.04	0.717	−0.47	<0.001	0.09	0.451

Models are adjusted for age, menopausal status, and all parameters of chronic systemic inflammation. BMI—body mass index, WC—waist circumference, RFM—relative fat mass, VAI—visceral adiposity index, WHtR—waist-to-height ratio, CRP—C-reactive protein, TNF-α—tumor necrosis factor alpha, IL-6—Interleukin-6, MetS—metabolic syndrome.

**Table 5:** Correlations between plasma adiponectin levels and cardiovascular risk factors.

	Premenopausal Women (*n* = 44)								Postmenopausal Women (*n* = 128)								All Women (*n* = 172)							
	CRP		TNF-α		IL-6		Adiponectin		CRP		TNF-α		IL-6		Adiponectin		CRP		TNF-α		IL-6		Adiponectin	
	r	*p*	r	*p*	r	*p*	r	*p*	r	*p*	r	*p*	r	*p*	r	*p*	r	*p*	r	*p*	r	*p*	r	*p*
BMI (kg/m^2^)	−0.07	0.636	−0.16	0.313	0.06	0.693	0.23	0.132	0.12	0.180	−0.01	0.916	0.20	0.025	−0.03	0.764	0.06	0.442	−0.05	0.534	0.16	0.033	0.05	0.502
WC (cm)	0.11	0.492	−0.03	0.859	0.25	0.096	0.23	0.125	−0.01	0.886	0.02	0.853	0.03	0.773	−0.07	0.452	0.02	0.798	0.01	0.923	0.07	0.349	0.01	0.933
SBP (mmHg)	0.26	0.094	−0.06	0.712	−0.11	0.465	−0.05	0.738	0.22	0.014	−0.11	0.231	0.15	0.082	−0.09	0.290	0.23	0.003	−0.10	0.210	0.11	0.146	−0.09	0.264
DBP (mmHg)	0.12	0.447	−0.01	0.961	−0.03	0.867	0.04	0.817	0.03	0.744	0.00	0.988	0.14	0.126	−0.01	0.871	0.05	0.507	0.00	0.989	0.10	0.197	0.00	0.990
FBG (mg/dL)	0.38	0.011	0.01	0.952	0.28	0.064	−0.47	0.001	0.42	<0.001	−0.07	0.408	0.18	0.047	−0.28	0.002	0.41	<0.001	−0.06	0.456	0.20	0.010	−0.32	<0.001
Insulin (mU/mL)	0.57	<0.001	0.06	0.694	0.13	0.409	−0.46	0.002	0.33	<0.001	−0.16	0.068	0.14	0.115	−0.45	<0.001	0.39	<0.001	−0.11	0.155	0.14	0.071	−0.45	<0.001
HDL (mg/dL)	−0.30	0.051	−0.21	0.173	−0.03	0.840	0.53	<0.001	−0.16	0.064	0.11	0.199	−0.07	0.415	0.46	<0.001	−0.20	0.007	0.04	0.644	−0.08	0.325	0.48	<0.001
LDL (mg-dL)	0.11	0.492	−0.07	0.663	−0.12	0.444	−0.27	0.073	−0.02	0.806	0.20	0.020	−0.02	0.819	0.11	0.209	0.01	0.869	0.14	0.068	−0.04	0.575	0.01	0.915
HOMA-IR	0.52	<0.001	0.03	0.826	0.22	0.151	−0.56	<0.001	0.46	<0.001	−0.16	0.072	0.20	0.022	−0.49	<0.001	0.48	<0.001	−0.10	0.176	0.20	0.009	−0.51	<0.001
CRP (mg/dL)	-	-	0.15	0.337	0.07	0.652	−0.16	0.293	-	-	0.11	0.210	0.20	0.025	−0.15	0.087	-	-	0.12	0.119	0.16	0.039	−0.15	0.045

r—Pearson’s correlation coefficient, BMI—body mass index, WC—waist circumference, SBP—systolic blood pressure, DBP—diastolic blood pressure, FBG—fasting blood glucose, HDL—high-density lipoprotein, LDL—low density lipoprotein, HOMA-IR—homeostasis model assessment of insulin resistance, CRP—C-reactive protein.

## Data Availability

The data presented in this study are available on request from the corresponding author. Our data supporting reported results can be found in the files of Pomeranian Medical University in Szczecin, Poland.

## Supplementary Materials

The following are available online at https://www.mdpi.com/article/10.3390/nu15122789/s1, Table S1: General characteristics of the population studied.
