# Supplementary material for: Relationships of Body Mass Index, Relative Fat Mass Index, and Waist Circumference with Serum Concentrations of Parameters of Chronic Inflammation
Source: Nutrients. 2023 Jun 18;15(12):2789. doi: 10.3390/nu15122789 (PMC10304469; doi:10.3390/nu15122789)
Supplement: Supplementary file 1 [file nutrients-15-02789-s001.zip › nutrients-2434084-supplementary.pdf]

Table S1. General characteristics of the population studied.

**Variables**			**Premenopausal women (n = 44)**		**Postmenopausal women (n = 128)**		**chi²**	***p*-value**
			**n**	**%**	**n**	**%**		
BMI	underweight	< 18.5	1	2.27	0	0.00%	4.018	0.259
	normal weight	18.5-24.9	14	31.82	32	25.00%		
	overweight	25.0-29.9	17	38.64	52	40.63%		
	obesity	≥ 30	12	27.27	44	34.38%		
WC	android body type	≥ 80 cm	11	25.00	29	22.66%	0.101	0.751
	gynoid body type	< 80 cm	33	75.00	99	77.34%		
RFM	average	25-31	6	13.64	14	10.94%	0.232	0.630
	obese	32+	38	86.36	114	89.06%		
VAI	no adipose tissue dysfunction (ATD)		35	79.55	94	73.44%	3.363	0.339
	mild ATD		1	2.27	14	10.94%		
	moderate ATD		4	9.09	12	9.38%		
	severe ATD		4	9.09	8	6.25%		
WHtR	slim	< 0.46	8	18.18	16	12.50%	2.471	0.650
	healthy	0.47-0.49	7	15.91	13	10.16%		
	overweight	0.50-0.54	11	25.00	33	25.78%		
	very overweight	0.55-0.58	6	13.64	23	17.97%		
	obese	0.55-0.58	12	27.27	43	33.59%		
Abdominal obesity		no	22	50.00	57	44.53	0.394	0.530
		yes	22	50.00	71	55.47		
General obesity		no	30	68.18	84	65.63	0.096	0.757
		yes	14	31.82	44	34.38		

BMI—body mass index, WC—waist circumference, RFM—relative fat mass, VAI—visceral adiposity index, WHtR—waist-to-height ratio.
